# Heterogeneity in the M. tuberculosis β-Lactamase Inhibition by Sulbactam

**DOI:** 10.21203/rs.3.rs-2334665/v1

**Published:** 2023-01-10

**Authors:** Marius Schmidt, Tek Narsingh Malla, Kara Zielinski, Luis Aldama, Sasa Bajt, Denisse Feliz, Brandon Hayes, Mark Hunter, Christopher Kupitz, Stella Lisova, Juraj Knoska, Jose Martin-Garcia, Valerio Mariani, Suraj Pandey, Ishwor Poudyal, Raymond Sierra, Alexandra Tolstikova, Oleksandr Yefanov, Ching Hong Yoon, Abbas Ourmazd, Petra Fromme, Peter Schwander, Anton Barty, Henry Chapman, Emina Stojković, Alexander Batyuk, Sébastien Boutet, George Phillips, Lois Pollack

**Affiliations:** Univ of Wisconsin, Milwaukee; University of Wisconsin Milwaukee; Cornell University; Northeastern Illinois University; Deutsches Elektronen Synchrotron; Northeastern Illinois University; Stanford University; SLAC National Accelerator Laboratory; Linac Coherent Light Source; Stanford Linear Accelerator Center; Center for Free-Electron Laser Science (DESY); Spanish National Research Council; Linac Coherent Light Source; University of Wisconsin Milwaukee; University of Wisconsin Milwaukee; Stanford pULSE Institute; CFEL; Center for Free-Electron Laser Science; Linac Coherent Light Source; University of Wisconsin-Milwaukee; Arizona State University; University of Winsconsin; Deutsche Elektronen-Synchrotron DESY; Deutsche Elektronen-Synchrotron DESY; Northeastern Illinois University; SLAC National Accelerator Lab; SLAC National Accelerator Laboratory; Rice University; Cornell University

## Abstract

For decades, researchers have been determined to elucidate essential enzymatic functions on the atomic lengths scale by tracing atomic positions in real time. Our work builds on new possibilities unleashed by mix-and-inject serial crystallography (MISC) ^1–5^ at X-ray free electron laser facilities. In this approach, enzymatic reactions are triggered by mixing substrate or ligand solutions with enzyme microcrystals ^6^. Here, we report in atomic detail and with millisecond time-resolution how the *Mycobacterium tuberculosis* enzyme BlaC is inhibited by sulbactam (SUB). Our results reveal ligand binding heterogeneity, ligand gating ^7–9^, cooperativity, induced fit ^10,11^ and conformational selection ^11–13^ all from the same set of MISC data, detailing how SUB approaches the catalytic clefts and binds to the enzyme non-covalently before reacting to a *tra*ns-enamine. This was made possible in part by the application of the singular value decomposition ^14^ to the MISC data using a newly developed program that remains functional even if unit cell parameters change during the reaction.

Beta(β)-lactamases are bacterial enzymes that provide multi-resistance to β-lactam antibiotics. They inactivate the β-lactam antibiotics by hydrolyzing the amide bond of the β-lactam ring^15,16^ (Extended Fig. 1a). Our study focuses on β-lactamase from *Mycobacterium tuberculosis* (*Mtb*)- the causative agent of tuberculosis. *Mtb* β-lactamase (BlaC) is a broad-spectrum Ambler class A ^17^ β-lactamase capable of hydrolyzing all classes of β-lactam antibiotics used for treatment of tuberculosis ^18,19^. In the latest report, the World Health Organization warned that multidrug-resistant tuberculosis is a public health crisis and a health security threat. In 2020, Tuberculosis was the 13th leading cause of death and the second leading infectious killer after COVID-19 (above HIV/AIDS), claiming 1.5 million lives worldwide ^20^. The rapid and worldwide emergence of antibiotic resistant bacteria, including *Mtb,* is endangering the efficacy of antibiotics, causing the Center for Disease Control (CDC) to classify several bacteria as urgent and serious threats ^21^. Research efforts focused on deciphering the molecular mechanism of antibiotic resistance within microbial pathogens such as *Mtb* can aid in novel-drug design, resistant to β-lactamase action, and therefore, contribute to managing this evolving crisis.

Structural information involving enzyme-substrate reaction intermediates is essential for understanding the mechanism of enzymes in action. Although several static X-ray structures of BlaC with various substrates have been determined ^18,22–27^ the reaction intermediates of β-lactamase reaction involving different antibiotic substrates remain mostly unknown. With time-resolved crystallography (TRX), conformational changes in biological macromolecules can be explored in real time ^28^. Once a reaction is initiated within the crystals, ensemble-averaged structures are obtained along a reaction pathway. In addition, the chemical kinetics that governs the biological reaction can be deduced ^14,29–32^. With mix-and-inject serial crystallography (MISC)^1^ single-turnover enzymatic reactions can be investigated in a time resolved manner. The substrate solution is mixed with enzyme micro-crystals before the mixture is injected into the X-ray beam. The reaction is triggered by diffusion of substrate and the resulting change is probed after a delay by short X-ray pulses ^3,4,33^. Using MISC, intermediates within the BlaC reaction with the third-generation cephalosporin-based antibiotic, ceftriaxone (CEF) were previously characterized from 5 ms to 2s ^1,33,34^.

Irreversible enzyme inhibitors represent potential new drugs in the fight against antibiotic resistance. Sulbactam (SUB), clavulanate and tazobactam, all suicide inhibitors with a β-lactam ring, irreversibly bind to β-lactamases and block their activity ^35,36^. As a result, the β-lactam antibiotics are protected from enzymatic degradation, which helps retain their efficacy. Due to its excellent solubility, SUB is a superb candidate for a MISC experiments involving BlaC.

Raman microscopy and mass spectrometry suggest a simplified mechanism for SUB binding to BlaC ^37,38^, shown in Fig. 1 and in Extended Data Fig. 1 with chemical structures. The first step is the formation of a reversible (non-covalent) enzyme inhibitor complex in the active site (E:I). Following this association step, the nucleophilic attack by the catalytically active serine on the β-lactam ring of the inhibitor leads to the formation of a short-lived, covalently bound acyl-enzyme intermediate (E-I). Further modifications of the chemical structure of the inhibitor lead to an inactivated enzyme (E-I*). The structures of intermediates shown in Extended Data Fig. 1 are relevant to the observed results presented in this paper within the measured timescale. SUB has also been described as a substrate of class A β-lactamases and can be hydrolyzed albeit at much slower rate than β-lactam antibiotics ^35,39^.

BlaC can be crystallized in a monoclinic space group with four subunits A - D in the asymmetric unit (Fig. 2a) ^1,40^. In this crystal form, the BlaC structure displays a large cavity of 30 Å diameter in the center. Additional cavities as large as 90 Å are identified within adjacent asymmetric units that allow easy diffusion of ligand molecules through the crystalline lattice. The E- I* structure of the BlaC-SUB has been determined at cryogenic temperature after soaking BlaC crystals for several minutes with SUB ^27,41–43^. More recently, pandey and colleagues captured an intermediate (presumably E:I) at a single time point (66 ms) after mixing BlaC crystals with SUB ^34^. Although subunits B/D displayed already a covalently bound adduct, an intact, non-covalently bound SUB was observed in subunits A/C (Fig. 2a). Subunits A/C are rather inactive, since they did not participate at all in the reaction with CEF in earlier experiments ^1,33^. Given the inactivity of subunits A/C, it was not clear whether the reaction with SUB takes more time to complete or proceed at all. Therefore, a time-series of MISC datasets is necessary, to further investigate both the binding of SUB inhibitor to and its subsequent reaction with the BlaC.

As in any time-resolved experiment, except in those performed on ultrafast time scales ^44–47^, multiple states can mix into any single time point observed during the reaction ^48^. As demonstrated for X-ray data ^14^ these mixtures can be characterized and potentially separated using the singular value decomposition (SVD). Within this context, SVD is an unsupervised machine learning algorithm ^49^ that can inform from time-resolved X-ray data the number of observable processes which is equivalent to the number of relaxation times and the number of structurally distinguishable time-independent reaction intermediates ^14^. In addition, it can provide information regarding the kinetic mechanism and the energetics of the reaction ^50–54^. SVD has never been applied to a time-series from a MISC experiment. Originally, the SVD method was developed to work with isomorphous difference maps assuming that the unit cells in the crystals do not change during a reaction. However, the unit cell parameters of the BlaC crystals vary after mixing (Extended Data Table 1). This makes a data analysis approach that relies on a stable volume of interest challenging. Therefore, a new suite of programs “pySVD4TX” was developed, that remains functional even when the unit cell parameters change.

## Binding Of Sulbactam

The four subunits of BlaC found in the asymmetric unit arrange in shape reminiscent to a torus (Fig. 2a). The alternating subunits are display similar binding kinetics while significantly differing from the adjacent ones. Here, subunits A and C share similarities. As do subunits B and D. However, there are distinct differences between these pairs. Whereas the catalytic clefts of subunits B and D are wide open, those of subunits A and C are partially occluded by the neighboring subunits (Fig. 2b; Supplementary Movies 1 and 2). particularly the residues Gln109^B/D^ and Gln108^B/D^ (the superscript B/D denotes residues from the neighboring subunits B and D, respectively) prevent substrate diffusion from the center of the torus, and Gln112^B/D^ and Arg173 block access to the active site from the exterior.

The reaction of SUB with BlaC was followed by difference electron density (DED) maps obtained at MISC delays (Δt_misc_) of 3 ms, 6 ms, 15 ms, 30 ms, 270 ms and 700 ms (Methods and Extended Data Table 1). At 3, 6 and 15 ms the DED features near the active site of subunit A are weak (Fig. 3a–c). Substantial displacements of the residues flanking the active site are observed (Extended Data Table 2; Supplementary Movie 1). particularly, the long side chain residues like Gln112^B^ and Arg173 (called here the guardian residues, Fig. 2b) move outward of the active site before relaxing back to original positions. Next to these residues at 3ms, almost 10 a Å way from Ser70, there are positive densities that are spatially more spread out than that of water. A SUB molecule can be placed in the electron density (Fig. 3a). However, refinement of the SUB is difficult (see Extended Data Fig. 2a for an explanation). At 6 and 15ms, density features appear closer towards Ser70 (Fig. 3b–c). These could indicate an initial trace of SUB molecules migrating to the active site after being held up by the guardian residues. Up to 15 ms, these densities are too weak that a SUB molecule can be placed with confidence.

At 30 ms, stronger DED features (max σ = 5.5) appear around 4.5 fr Å om the catalytically active Ser70 (Fig. 3d). An intact sulbactam can be modelled which reproduces and corroborates the findings at 66 ms obtained from an earlier experiment ^34^. The β-lactam ring is oriented away from the Ser70. Between 66 ms and 240 ms, the SUB must rotate so that the β-lactam ring is positioned towards the Ser70 at which point the nucleophilic attack occurs (Extended Data Fig. 1a). At 240 ms, the elongated DED feature that originates from the Ser70 directly supports the presence of a covalently bound *trans*-enamine (TEN) (Fig. 3e). The BlaC-TEN adduct structurally relaxes until 700 ms, the final time point in the time series (Fig. 3f). These snapshots of the reaction in progress were assembled to a movie (Supplementary Movie 1) of an enzyme in action.

In subunit B, there is no evidence of an intact SUB that accumulates in the active site (Fig. 3g–h). Even the features that appeared near Arg173 in subunits A/C are not present. However, at 15 ms, the presence of strong DED that extends from the Ser70 supports a covalently bound TEN (Fig. 3i, Extended Data Fig. 2b). The occupancy refined to 85% in B and 86% in D. This suggests the reaction is close to completion. At later time points, no large changes in the structure of the BlaC-TEN complex are apparent. (Fig. 3j–l). However, in subunits A/C, the reaction keeps progressing and structural changes of both the entire enzyme and the TEN are observed (Supplementary Movie 1).

## Temporal Variation Of Difference Electron Density

The SVD analysis is required to identify the number of intermediates as well as the relaxation times from the time-series of DED maps ^14^ (see Methods). The right singular vectors (rSVs) obtained from the SVD analysis plotted as a function of MISC time delays represent the temporal variation of the reaction. It is important that the relaxation processes inherent to each rSV are accurately determined. The slow initial progress and sudden increase of the magnitude of DED values in the active sites require an appropriate function that could account for this behavior. An excellent fit was obtained by Eq. 2 which consists of a (step-like) logistic function that accounts for the steep first phase and an exponential saturation component. Detailed values of the parameters of Eq. 2 are shown in the Extended Data Table 3.

The first two significant rSVs for each subunit are shown by blue and red squares respectively (Fig. 4a–d). They allow for the determination of relaxation times τ_1_ and τ_2_ by fitting Eq. 2, shown by their respective colored solid line. The rest of the rSVs, shown by colored diamonds in Fig. 4a, are distributed closely around zero and do not contribute to the subsequent analysis. The process with relaxation time τ_1_ results from the first appearance of DED in the active sites. In subunits A/C, this process reflects the appearance of the non-covalently bound SUB which occurs at around 23.5 and 24.5 ms, respectively. In subunits B/D, a covalently bound TEN is observed at 9.6 ms and 10.2 ms respectively after mixing. In subunits A/C, the process τ_2_ results from the transformation of intact SUB to covalently bound TEN which happens approximately 75 ms and 90 ms after mixing. In contrast, in subunits B/D TEN is present already during the first phase (Fig. 3g–l; Extended Data Fig. 2e-h) and no further chemical modification of the TEN is observed. Despite this, a second relaxation process is also observed which coincides with the SUB to TEN formation in subunits A/C.

## Inhibitor Diffusion

The kinetics of SUB binding to subunits B/D is evaluated first, since it allows for an estimate of the ligand concentration in the unit cell that is required to analyze the observations for subunits A/C. The total concentration of BlaC monomers in the crystals (E_free_) is ~ 16 mM. If only subunit B is considered, the effective [E_free_] is 4 mM. The apparent diffusion time of SUB into the microcrystals is ~ 7 ms (Extended Data Table 4). It needs to be pointed out that MISC does not directly measure diffusion inside crystals.

Instead, free ligand concentrations, and related to them the diffusion time, are estimated only indirectly through the rate equations (Eqs. 3 and 4) which ultimately must reproduce the observed (refined) ligand occupancies ^34^. The unknown parameters in these equations must be varied until the concentration of species [E:I] and [E-I*] best match their respective occupancy values (Fig. 5a). Additionally, relaxation times derived from the concentration profiles of intermediates must also agree with those obtained from SVD analysis of the DED maps (compare Extended Data Tables 3 and 4). The concentrations of the free SUB within the microcrystals rise slightly slower than the values estimated in the central flow of the injector (Extended Data Table 4, compare Fig. 5a blue solid line and blue squares). The use of a logistic function (Eq. 3) that describes the ligand increase in the unit cell is justified in particular by (i) the steep first phase observed in the rSVs (Fig. 4), but also by (ii) the very rapid increase of the ligand in the inner flow of the injector constriction where saturation occurs already after 15 ms. The covalently bound TEN accumulates rapidly with a characteristic time of 11.5 ms (Extended Data Table 4) which is in line with the result from the SVD analysis (~ 10 ms). No additional intermediate is observed.

In subunits A/C, the apparent diffusion time of SUB necessary to reproduce the occupancies of the non-covalently bound intermediate is 20 ms (Fig. 5b and Extended Data Table 4). This is much longer than that observed in subunits B/D (7 ms). This lag can only be explained by a restricted access to the active site. The guardian residues open the active site after about 6 ms (Supplementary Movie 2) which corresponds to about 35 mM outside ligand concentration (Fig. 5a). Entry to active site is controlled by an additional rate coefficient, k_entry_. k_entry_ was modelled (Eq. 5) by an exponential function that depends on the (time-dependent) concentration difference Δ*I(t) = I*_*out*_*(t) — I*_*in*_*(t)* between the outside and the inside of the active site, respectively, and a characteristic concentration difference ΔI_c_ set to 25 mM (Extended Data Table 4). I_out_ is the SUB concentration in the unit cell, which is known from the substrate binding kinetics to subunits B/D (see above). After a delay of ~ 20 ms, SUB enters the active sites of subunits A/C and I_in_ rapidly increases. On the same time-scale the non-covalent E:I intermediate accumulates (compare τ_1_ in Fig. 4a, c and Fig. 5b). The non-covalently bound SUB triggers the next step of the reaction which results in a covalently bound TEN. The concentration of TEN ([E-I*]) starts dominating [E:I] at around 90 ms (Fig. 5b) which is in agreement with τ_2_ obtained from the SVD analysis (Fig. 4a, c). By 240 ms, more than 9 *%* of the crystal is occupied by TEN (Fig. 5b).

## Discussion

While it is established that all the subunits take part in the reaction despite the heterogeneity, they do so at different rates. In subunits A/C, BlaC is inhibited by SUB via a two-step mechanism. The non-covalent intermediate can accumulate since the SUB is not properly oriented (Fig. 3d) and can react to TEN only after an additional time delay. SUB displacements will be restricted by interactions with the surrounding residues (Extended Data Table 2). To react with the active Ser70, the SUB must re-orient to expose the β- lactam ring towards the serine. The catalytic opening of the β-lactam ring and the unfurling of the thiazolidine ring all happen in succession inside the narrow reaction center cavity. This severely lowers the rate of TEN formation.

**Figure 1:**
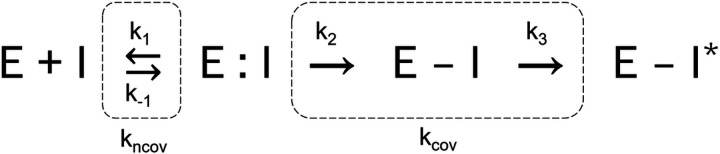
A simplified two-step mechanism of BlaC inhibition by sulbactam. The first step is the formation of the non-covalent enzyme inhibitor complex whose rate of formation depends on the concentration of the inhibitor in the unit cell and the rate coefficient (k_ncov_). The nucleophilic attack by the active serine opens the lactam ring of SUB leading to the formation of acyl-enzyme intermediate (E-I). The E-I intermediate does not accumulate to become observable. The next step is the irreversible inhibition of enzyme by the chemically modified inhibitor (E-I*) which depends on the apparent rate coefficient (k_cov_).

In subunits B/D the covalent binding of SUB can apparently be explained by a one-step mechanism. However, the two-step mechanism described in Fig. 1 is also consistent with the observations when the non-covalent binding of substrate to the enzyme is much slower than the formation of TEN. By applying the two-step mechanism (Eq. 4), a k_ncov_ value of ~ 1.5 mM^−1^ s^−1^ and a large k_cov_ value of ~ 8000 s^−1^ are estimated (Extended Data Table 4) so that the non-covalently bound E:I complex does not accumulate. The open binding pocket accommodates for large chemical and structural changes which result in the large k_cov_. The rate determining step is controlled by k_ncov_ although the non-covalent BlaC-SUB complex never accumulates. In a one-step scenario (the free SUB reacts directly to TEN), the pseudo one-step rate coefficient would have to be the same as the k_ncov_, which was confirmed with a separate calculation (not shown). The two-step mechanism explains the enzymology in the active sites of all subunits in a consistent way.

A second relaxation phase is observed in the SVD analysis of DED maps from subunits B/D (Fig. 4b and d), although the reaction to TEN has already taken place. It appears as if this is a result of the continuing reaction in subunits A/C and, related to this, an ongoing relaxation of the entire protein structure (Supplementary Movie 1). The relaxation times, τ_2_, of the second relaxation phase in all four subunits are all tied together between 80 ms and 90 ms (Extended Data Table 3), which is an indication for a cooperative behavior of all subunits in BlaC. This observation would have been obscured without the prowess of SVD which can track DED values across the entire reaction.

## Rapid Reaction With Sulbactam In BlaC Microcrystals

By taking into account the molecular volume that is enclosed by the van der Waal’s surface, SUB (181.7 Å^3^) is 2.5 times smaller than CEF (444.9 Å^3^). For CEF, 50% occupancy of the enzyme substrate complex has been observed at Δt_MISC_ = 5 ms in subunits B and D ^34^. Due to the smaller size, SUB should diffuse faster into the crystals than CEF and the signature of an enzyme inhibitor complex is expected to appear already at the earliest time points (3 ms and 6 ms). However, the first event that could be identified in the DED maps is the appearance of the TEN at 15 ms in subunits B/D (Fig. 3i). The concentrations of SUB at the active site of subunits B/D, which allow direct, unrestricted access, can be taken as an estimate of the SUB concentration inside the BlaC crystals. The slight lag between the estimated SUB concentration in the inner flow of the injector constriction and the concentrations in the crystals at early time points (Fig. 5a, compare blue squares with the blue line) is expected, since diffusion in BlaC crystals is slowed down in crystals ^34^ compared to water. The apparent diffusion time (7ms, Extended Data Table 4) of the SUB is almost the same as that for the antibiotic substrate CEF (~ 5 ms)^34^. Still, contrary to expectations based on the size of the SUB, no electron density has been present at the earliest time points. This can be explained by the smaller second order binding coefficient, k_ncov_ (1.5 mM^−1^ s^−1^ for SUB compared to 3.2 mM^−1^ s^−1^ for CEF), that prevents the accumulation of electron density at earlier times.

The characteristic times observed in both classes of subunits for the formation of the covalently bond TEN species (around 10 ms and 90 ms, respectively, Fig. 5) are quite fast in comparison to earlier suggestions that the reaction might take minutes to complete ^27,41^. The fast reaction provides an advantage when β-lactam substrates and the SUB inhibitor are competing for the same active site. For example, the non-covalent enzyme-substrate complex with CEF persists for up to 500 ms ^33^. During this time, the CEF can leave the enzyme and be replaced first competitively and then irreversibly by a quickly reacting SUB molecule. Since the inhibitor competes with co-administered antibiotics for the active site of BlaC, one can imagine that the covalent bond formation with an inhibitor must occur as fast as possible to effectively eliminate β-lactamase activity in the presence of substrate. This is in addition corroborated by Jones and coworkers who reported that SUB has a ten times higher affinity and binding constant for plasmid mediated class A β-lactamases compared to cefoperazone ^55^, which is a third-generation cephalosporin-based antibiotic similar to CEF.

## Ligand Gating, Induced Fit And Conformational Selection

**Figure 2:**
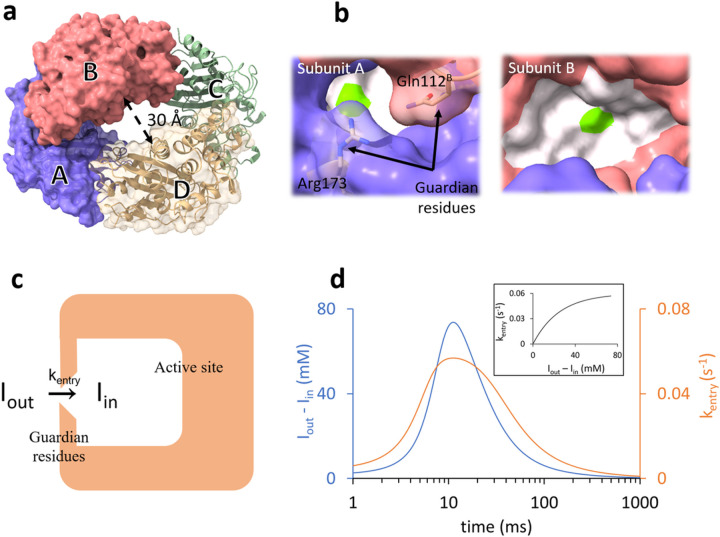
Structure of BlaC and the gating mechanism. (a) Subunits A - D in the asymmetric unit are marked and shown by blue, red, green, and yellow respectively. (b) Binding pockets of subunit B (top) and subunit A (bottom). The active site is represented by the white surface. The position of the catalytically active serine is marked in green. The access to the active site in subunit B is wide open. The entrance to the active site of subunit A is partially occluded by two residues (Gln112^B^ and Arg173) called the guardian residues. (c) Simplified scheme depicting the delayed entry of sulbactam into the active site through the guardian residues in subunits A/C. (b) Time-dependence of the concentration difference (blue line) and of the rate coefficient k_entry_ (orange line). Inset: The dependence of k_entry_ on the concentration difference.

**Figure 3:**
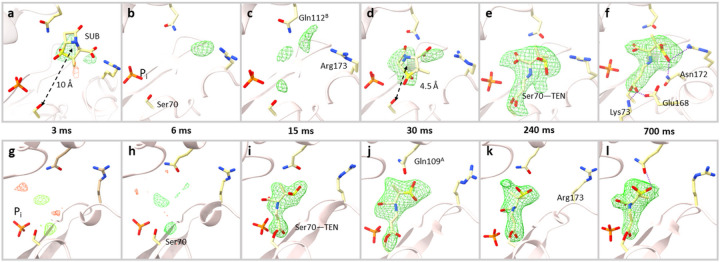
Difference electron density maps at the active site of subunit A and subunit B (contour level ±3σ). *Subunit A top row:* (a) At 3ms, weak densities can be identified at the entrance of cavity between Gln112^B^ and Arg173 and a SUB placed there. (b) At 6ms, very weak density is observed. The phosphate molecule (P_i_) near the active site is marked. (c) At 15ms, difference density features are identified closer to the catalytically active residue Ser70. The guardian residues (Gln112^B^ and Arg173) that are located at the entrance to the binding pocket are marked. (d) At 30 ms a strong DED feature appears within the active site. An intact SUB molecule is placed there. (e) At 240 ms, the SUB has reacted with Ser70 to form TEN giving rise to an elongated density. (f) At 700 ms, the elongated density of the TEN is fully developed. Additional hydrogen bonds between the TEN and other side chains are shown. *Subunit B, bottom row:* (g-h) At 3 and 6ms, no interpretable density was present in the catalytic center. (i) At 15ms, the SUB has already reacted with Ser70 to from TEN. (j-l) TEN densities as observed at Δ_misc_ from 30 ms to 700 ms. Gln109^A^ and Arg173 are marked in j and k, respectively.

**Figure 4:**
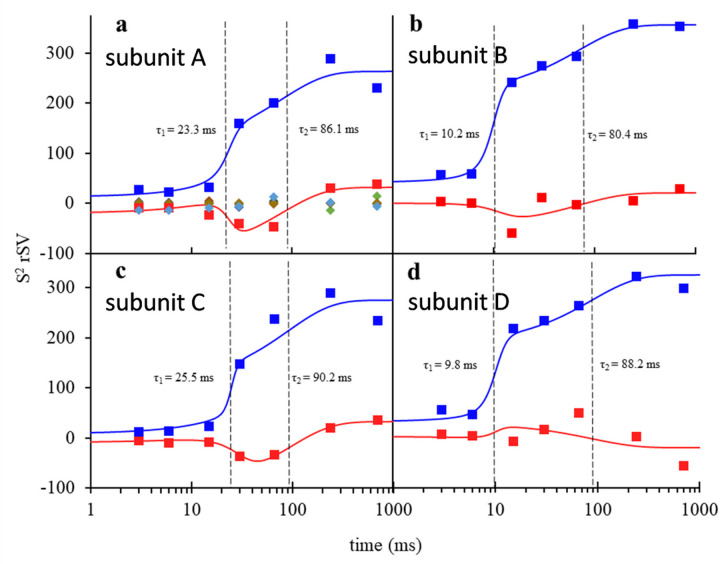
Right singular vectors (rSVs) derived from a singular value decomposition of the time-dependent DED maps in the active sites of the BlaC. (a) Right singular vectors plotted as a function of Δt_misc_ for subunit A. The first and second significant rSV are shown by blue and red triangles respectively. Solid colored lines are the result of a global fit of Eqn. 2 to the significant rSVs. The colored diamonds represent insignificant rSVs. (b-d) Significant rSVs plotted as a function of Δt_misc_ for subunits B, C and D respectively. Colors and lines as in panel (a). The vertical dashed black lines in all panels denote the relaxation times τ_1_ and τ_2_ that result from the fit. For subunits A and C, τ_1_ belongs to accumulation of intact SUB in the active site, and τ_2_ corresponds to the formation of the covalently bound TEN. For subunits B and D, τ_1_ denotes the time when the reaction to TEN occurs and τ_2_ indicates a second relaxation phase.

**Figure 5:**
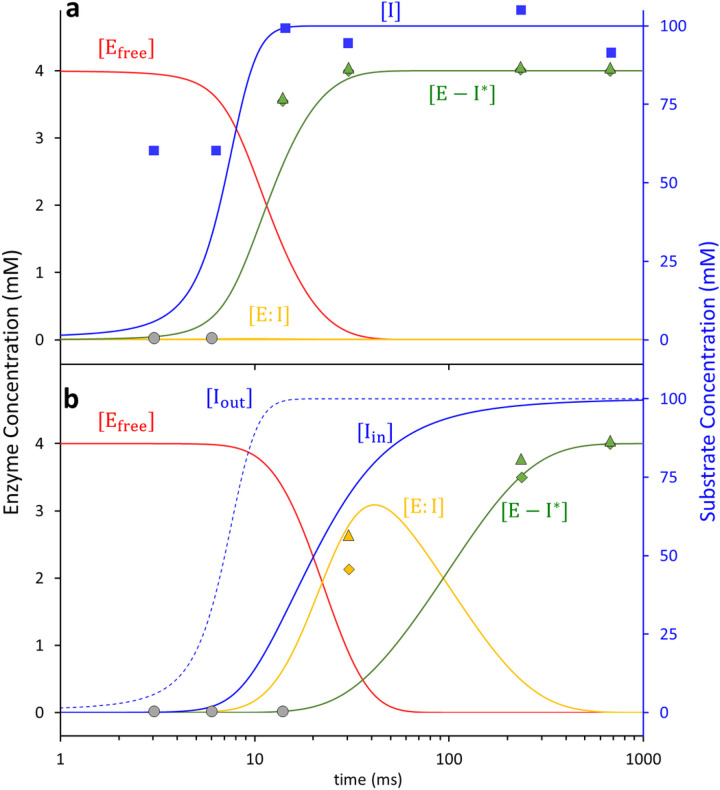
Calculated concentration profiles of reactants and products in the active sites of BlaC compared to corresponding observables. (a) Subunits B/D. Blue line: free SUB concentrations [I] in the unit cell. Blue squares: SUB concentrations in the central flow of the injector. Red line: time-dependent concentrations of the free BlaC [E_free_]. Orange line: concentrations of the non-covalently bound SUB [E:I] intermediate (not observable). Green line: concentrations of the covalent enzyme-inhibitor complex TEN [E-I*]. Green triangles and diamonds: concentrations of E-I*, derived from refined ligand occupancy values in subunits B and D, respectively. Grey circles: SUB cannot be detected near the active sites. (b) Subunits A/C. Blue dotted line: free SUB concentrations [I] in the unit cell. Blue line: SUB concentrations [I_in_] in the active site (note the delay relative to subunits B/D). Red line: time-dependent concentration of the free BlaC [E_free_]. Orange line: concentrations of the non-covalently bound SUB [E:I] intermediate. Green line: concentrations of the covalent enzyme-inhibitor complex TEN [E-I*]. Orange and green triangles and diamonds: concentrations of E:I and E-I* derived from refined ligand occupancy values in subunits B and D, respectively.

Our results show that ligand binding to enzymes may be more complicated than initially thought ^27,37,38^. Only after a delay the ligand penetrates into the active sites of subunits A/C controlled by the guarding residues (Fig. 5b). The narrow entrance to active site (Fig. 2b; Supplementary Movie 2), and the displacements of the guardian residues (Extended Data Table 2; Supplementary Movies 1 and 2) are reminiscent of a substrate tunneling-and-gating ^7–9^ like mechanism which has not yet been discovered in published structures of BlaC. Supplementary Movie 2 shows, that the movement of the guardian residues open the entrance, thereby controlling the access of ligand. More work is needed to determine the mechanism that drives the displacement of these residues. As these displacements were not observed when reacting with CEF ^33,34^, an allosteric mechanism that links the position of the guardian residues to the covalent binding of SUB in adjacent subunits is unlikely. However, electrostatic interactions ^56^ of the negatively charged sulbactam with the positively charged Arg173 or even polar interaction with the Gln112^B,D^, respectively, may plausibly induce these structural changes.

At Δt_MISC_ of 30 ms and 66 ms ^34^ SUB occupies the active sites of subunits A/C without reacting with Ser70. During this time conformational changes of the BlaC are apparent (Supplementary Movie 1) to accommodate the SUB. This resembles an induced fit ^10^. The unfavorable SUB orientation prevents the direct attack of the active Ser70 towards the β-lactam ring. Ser70 can react with the β-lactam ring only after a rotation of the SUB. Fluctuations of the Ser70 towards the SUB carbon C_7_ will then lead to a very short lived, indiscernible “transition state like” structure E-I with a covalent bond between the SUB and BlaC. This is a different form of conformational selection ^12,13^, in a sense that there is not a particular “preferred” protein conformation that reacts with a substrate, but here, a particular (active) ligand orientation is required and “selected” by the enzyme for further reaction. It is the rate of the reorientation (rotation of the SUB) that seems to control the rate of this reaction. Once a favorable orientation is reached, further reaction to TEN is instantaneous on the timescale of the observation (90 ms) (Extended Data Table 4). This informs the design of improved (faster) inhibitors that consist of symmetric active moieties ^57,58^ or are engineered to enter the active sites in the correct orientation.

In subunits B/D, the inhibitor is brought in rapidly by diffusion and reacts instantaneously (< 1.5 ms) on the timescale of observation (> 15 ms). Neither an induced fit nor conformational selection can be observed or distinguished which previously led to intense discussions for other enzymes ^11^. The active site structures relax in unison with the extent of covalently bound inhibitor in all subunits as explained above.

### The Fate of the Trans-Enamine

It has been proposed that on longer timescales (>30 min) a second nucleophilic attack by a nearby serine can occur in other, structurally closely related Ambler Class A β-lactamases ^35^. This serine (Ser128 in BlaC) may react with the C5 position of the TEN (Extended Data Fig. 1). This is followed by the loss of the opened thiazolidine ring fragment (Extended Data Fig. 1d). A covalent bond may be formed between C5 and Seri 28 of BlaC (Extended Data Fig. 2c) leading to the prolonged inhibition of the enzyme ^35,41^. It has also been suggested that only the transient inhibition by TEN is responsible for the medical relevance of SUB as any reaction that lasts longer than one hour is irrelevant due to the bacterial lifecycle of ~ 30 minutes ^41^. However, the life cycle of *Mtb* is around ~ 20 hours ^59^. The permanent inhibition achieved only after the second nucleophilic attack might be the ultimate factor for SUB’s clinical usefulness in fighting antibiotic resistance in slow growing bacteria like *Mtb.* Inspection of the soaked structure may give an answer. Covalently bound TENs were observed in all four subunits when BlaC was soaked with SUB for 3 hours (Extended Data Fig. 2e-h). The B-factors of the fragment beyond N4 that would be cleaved off (displayed in pale colors in Extended Data Fig. 2c) are consistently higher by 20 Å^2^ than that of the part which would form the cross-linked species. However, it is more plausible that higher B-factors are caused by the dynamic disorder of the long TEN tail and not by the presence of mixture of intact and fragmented TEN. There is no clear evidence of TEN fragmentation, and TEN remains the physiologically important species for BlaC inhibition for hours.

A phosphate group binds to a specific site immediately adjacent to the active serins 70 and 128 (Extended Data Fig. 2d) with multiple hydrogen bonds to surrounding residues. This location appears to be conserved among all published BlaC structures where others have also reported sulfate and acetate molecules in the same position (Extended Data Fig. 2d) ^27,60–62^ Naturally occurring compounds with phosphate like group, for instance adenosine phosphates, might also interact with BlaC. β-lactamase production increases in some bacteria grown in a phosphate enriched medium ^63^ while phosphate can also promote the hydrolysis of the clavulanate inhibitor by BlaC ^62^. More structures are required after soaking with high SUB concentrations for longer periods of time, perhaps days, to observe potentially fragmented TEN. Since the phosphate is replaceable ^1,33,34^, the TEN might indeed react further. Larger inhibitors like tazobactam and clavulanic acid might also be able to displace the phosphate molecule directly. Recently, *trans-enamine* intermediates of tazobactam were identified in the serine β-lactamase TEM-171 at a position similar to that of TEN in BlaC and at another that is occupied by the phosphate in BlaC ^64^. The diverse chemistry that is already observed very early on in BlaC may extend to much longer time scales.

There are other classes of β-lactamases that are more concerning than BlaC such as the metallo β-lactamases (MBL). They are capable of hydrolyzing almost all clinically available β-lactam antibiotics and inhibitors ^65,66^. Similar work to the one presented here and earlier ^33,34^ could be performed on MBLs to gain more structural insight into their catalytic mechanisms. Time-resolved pump-probe crystallographic experiments using a caged Zn molecule ^67^ already show how the antibiotic moxalactam is inactivated by a MBL on a timescale longer than 20 ms. To characterize the important substrate binding phase on single ms, and even sub-ms time scales, it would be desirable to follow this or a similar reaction as well as the binding of a MBL inhibitor with MISC.

## Conclusions

MISC is a straightforward way to structurally study enzyme function. Reactions are visualized in real time as movies of the enzyme in action. Our results give insight how the shape of the active site determines rate coefficients and reaction mechanisms of a biomedically relevant reaction. From the MISC data, diffusion times and rate coefficients can be estimated by applying informed, chemically meaningful constraints and tying the analysis to observed occupancy variations of transient enzyme-inhibitor complexes in the different subunits. The key advantage of MISC is that it provides at the same time a localized view onto processes that unfold in individual subunits and a global perspective of the behavior of the entire molecule with near atomic precision. High repetition rate XFELs ^68,69^ and upgraded synchrotron light sources ^70,71^, will facilitate the collection of time-series that consists of very closely spaced MISC time-delays. A global evaluation of these time-series assisted by a principal component analysis such as the SVD and other machine learning techniques ^47,49^ will provide a detailed and direct view into enzyme catalysis and inhibition.

## Methods

### Singular value decomposition

A kinetic analysis was performed by the application of the singular value decomposition (SVD) to the X-ray data ^14^ using the DED_omit_ maps (for data collection and difference map calculation, see the Supplementary Information, SI). A region of interest (ROI) was determined that corresponds roughly to the volume of an individual active site selected from the four subunits in the asymmetric unit. The ROI covers the volume occupied by the SUB and TEN ligands and the entire side chain of Ser70. It touches the terminal atoms of residues Lys 73, Glu168, Thr239, Asn172, Arg173, Ser128, Ser102 and Gln112 (from B/D) in the case of subunits A/C. Difference electron density values found in grid points (voxels) of the ROI were assigned to a m-dimensional vector. N = 6 of these vectors were obtained for measured Δt_misc_ and assembled to a m x n dimensional matrix **A**, called the data matrix. SVD is the factorization of matrix **A** into three matrices: **U, S** and **V**^**T**^ according to

1 $$A=U\cdot S\cdot V^{⊤}$$

The columns of the m × n matrix **U**, are called the left singular vectors (ISVs). They represent the basis (eigen) vectors of the original data in data matrix **A. S** is an n × n diagonal matrix, whose diagonal elements are called the singular values (SVs) of A. These non-negative values indicate how important or significant the columns of **U** are. The columns of the n × n matrix **V**, called the right singular vectors (rSVs), contain the associated temporal variation of the singular vectors in **U. S** contains n singular values in descending order of magnitude.

The number of significant singular values and vectors can inform how many kinetic processes can be resolved ^14,72^. The SVD results can then be interpreted by globally fitting suitable functions to the rSVs, that can consist, in the simplest form, of sums of exponentials. The rSVs contain information on the population dynamics of the species involved in the mechanism ^14,72^.

The earlier program SVD4TX ^14^ and a newer version ^73^ could not be applied to X-ray data when large unit cell changes occur during the reaction since these implementations relied on a region of interest that is spatially fixed. This is not given when the unit cell changes. In order to accommodate changing unit cells, a new approach was coded by a combination of custom bash scripts and python programs described below.

### Adapting SVD for MISC Datasets with Changing Unit Cell parameters

The DED map are calculated (Supplementary Methods) in the CCp4 file format ^74^ and cover the entire unit cell of the crystal. The maps are represented by a three-dimensional (3D) array with m_x_, m_y_ and m_z_ grid points for each unit cell axis, respectively. Each 3D grid point (voxel) contains the magnitude of the difference electron density at that given position (Extended Data Fig. 3 a). In such a DED map, positive features indicate regions where atoms have shifted away from their position in the reference model. Negative features are then found on top of the atoms in the reference model. Most of the map contains only spurious noise except in ROIs such as the active sites where larger structural changes are expected due to the binding or dissociation of a ligand (Extended Data Fig. 3 b). The noise within the majority of the difference map would interfere with the SVD analysis. To avoid this, a ROI was isolated individually for each subunit and an SVD performed only on the DED within. When multiple active sites are present, each active site can be investigated separately. Extended Data Fig. 4 shows a flow chart of the steps required to prepare the data matrix **A**. The steps are described in detail below.

#### Step 1:

The coordinates of the atoms of the amino acid residues and the substrate of interest are specified in a particular subunit. This defines the ROI. For the present work, four different ROIs were defined, one for each subunit A to D, respectively, and investigated separately.

#### Step 2:

A mask is calculated that covers the selected atoms plus a margin of choice (Extended Data Fig.3 b). The density values outside of the mask are set to 0 while the ones inside are left unchanged. This results in a masked map with the dimensions of original map with density values present only around the emerging DED in the active site (Extended Data Fig.3 c). This mask was evolved later (after step 4) by allowing only grid points that contain DED features greater or smaller than a certain sigma value (for example, plus or minus 3 σ) found at least in one time point ^14^.

When the unit cell parameters do not change during the reaction, the difference maps at all time points will have the same number of voxels and the voxel size is also constant. However, once the unit cell dimensions change, either the voxel size will change, if the number of voxels is kept constant, or the number of voxels will change, if the voxel size is kept constant. If the voxel size changes, the DED value assigned to each voxel position will also change which will skew the SVD analysis. If the voxel numbers change, the SVD algorithm will fail as it requires that all the maps are represented by arrays of identical sizes. Accordingly, both conditions, (i) a constant voxel size and (ii) a constant number of grid points in the masked volume must be fulfilled when the unit cell changes.

#### Step 3:

In order to fulfill (i) the total number of grid points in the DED map is changed proportionally to the unit cell change. When the volume of the ROI is not changed, condition (ii) is automatically fulfilled, and a suitable data matrix **A** can be constructed. However, when the unit cell parameters change, the ROI is also changing position. This must be addressed in addition.

#### Step 4:

A box is chosen that will cover the density that was just masked out (Extended Data Fig.3 d). The box will include the ROI which is saved as a new map. The size of the box must be large enough such that the ROIs can be covered at all time points. The box must be calculated with reference to a stable structure (usually the protein main chain). As the protein chain displaces as a result of the change of the unit cell, the box will also move accordingly to cover the correct ROI (Extended Data Fig.3 e). As mentioned, the DED within the moving box can be used to evolve the mask that defines the final ROI as indicated in step 2.

#### Step 5:

All m voxels in the evolved mask are converted to a one-dimensional (1D) column array, a vector in high (m) dimensional space. How the conversion is achieved does not matter as long as the same convention is applied to all the n maps. N of the m-dimensional vectors are arranged in ascending order of time to construct the data matrix **A**.

#### Step 6:

SVD is performed on matrix **A** according to Eqn. 1.

#### Step 7:

Trial functions are globally fit to the significant rSVs to determine relaxation times and the minimum number of intermediates involved in the reaction (see e.g., Ihee et al., 2005^53^).

### Global fit of the Significant rSVs

For a simple chemical kinetic mechanism with only first-order reactions, relaxations are characterized by simple exponentials ^48^. For higher order reactions, the rSVs have to be fitted by suitable functions which must explain the changes of the electron density values in a chemically sensible way ^14,29,75^. In our case, the significant rSVs were fitted by Eq. 2 which, apart from a constant term, consists of a logistic function that accounts for abrupt changes of the electron densities observed in the active sites, and an additional saturation term. Further, the fit was weighted by the square of the corresponding singular values S_i_.


2 $$S_{i}^{2}rSV_{i}(t)=A_{0,i}+\frac{A_{1,i}}{1\;+\;e^{-\lambda \left(t-\tau_{i}\right)}}+A_{2,i}\left(1-e^{-\frac{t}{\tau_{2}}}\right)$$

While the amplitudes A’s are varied independently for each significant rSV_i_ (i = 1 ...n), the parameter λ and the relaxation times, τ_1_ and τ_2_ are shared globally. The number of relaxation times (here 2) is equal to the number of significant rSVs and to the number of distinguishable processes.

### Species Concentrations

The diffusion of SUB molecules into the BlaC crystals and subsequently into the active sites triggers the reaction. Concentrations of SUB in the central flow were estimated according to Calvey et al., 2019 ^76^. At the longest Δt_misc_= 700 ms, the SUB concentration was 100 mM, which was used as the maximum ligand concentration for all calculations. The resulting evolution of concentration of SUB at the active sites was modeled by Eq. 3.

3 $$I(t)=\frac{I_{\max}}{1\;+\;e^{-\mu \left(t-t_{0}\right)}}$$

I(t) is the concentration of SUB at the active sites averaged over all unit cells in the crystal as a function of time and I_max_ is the maximum (100 mM) SUB concentration (Extended Data Table 5). Eq. 3 is a logistic function where μ is the growth rate and t_0_ is the midpoint value of the growth.

Once the SUB molecule reaches the active site of BlaC, the first step is the formation of a non-covalent enzyme inhibitor complex (E:I) (Fig. 1). The process depends on the free BlaC concentration inside the crystal, and the rate coefficient for non-covalent complex formation (k_ncov_). This step is usually reversible defined by both the forward rate coefficient (k_1_) and the backward rate coefficient (k_−1_). However, the mounting concentrations of inhibitor inside the crystals forces more molecules towards the active site. At least initially, the binding rate depends on k_1_ alone. The non-covalent E:I complex is the reactant for the next phase of the reaction where the β-lactam ring opens. The resulting covalently bound acyl-enzyme complex (E-I) (Fig. 1) is so short lived that it never accumulates in the timescale of the measurement. The SUB undergoes rapid modification, and a product is formed where the enzyme is covalently bound to the irreversibly modified inhibitor (E- I*). k_cov_ is the apparent rate coefficient which describes the velocity of EI* formation directly from the E:I complex (Fig. 1). Ligand concentrations were determined by numerically integrating the following rate equations that describe the mechanism in Fig. 1.

4 $$d[E:I]=\left[E_{free}\right]\left(t_{i}\right)\cdot [I]\left(t_{i}\right)\cdot k_{n\;\text{cov}}\cdot dt\;\;\left[E_{free}\right]\left(t_{i+1}\right)=\left[E_{free}\right]\left(t_{i}\right)-d[E:I]\;\;d[E-I*]=[E:I]\left(t_{i}\right)\cdot k_{\text{cov}}\cdot dt\;\;[E-I*]\left(t_{i+1}\right)=[E-I*]\left(t_{i}\right)+d[E-I*]\;[E:I]\left(t_{i+1}\right)=[E:I]\left(t_{i}\right)+d[E:I]-d[E-I*]\;\;t_{i+1}=t_{i}+dt$$

d[E:I] is the change in concentration of the non-covalent BlaC-SUB complex at any given time (t_i_) and depends on the free enzyme concentration, [E_free_], the second order rate coefficient of non-covalent binding (k_ncov_), and the inhibitor concentration, [I]. [I] is calculated from Eq. 3. As the concentration of [E:I] increases, [E_free_] decreases. d[E-I*] is the increase of the covalently bound TEN. It depends on the available concentration of the non-covalent BlaC-SUB complex [E:I], and the rate coefficient k_cov_. [E:I] decreases by the same rate [E- I*] increases.

The increase of the SUB concentration in the active site (I_in_) is delayed relative to that of the SUB concentration in the unit cell (I_out_). To account for this delay, the rate coefficient that determines the entry into the active site (k_entry_, Fig. 2 c) is assumed to be dependent on the concentration difference Δ*I(t) = I*_*out*_*(t) — I*_*in*_*(t)* between outside and inside the active site and a characteristic difference Δ*I*_*C*_ . It is modeled by an exponential function

5 $$k_{entry}=k_{\text{max},\text{entry}}\left(1-\exp^{-\frac{\Delta I(t)}{\Delta I_{c}}}\right)$$

The relevant SUB concentrations within the active site (I_in_) are generated by solving the following rate equation:

6 $$\begin{array}{l} dI_{in}=k_{entry}\cdot I_{out}\cdot dt\\ I_{in}\left(t_{i+1}\right)=I_{in}\left(t_{i}\right)+dI_{in}. \end{array}$$

Eqn. 6 is used in lieu of Eq. 3 to calculate the relevant inhibitor concentration: I_in_(t) is fed as [I](t) to Eq. 4 to calculate the concentrations of the non-covalently and covalently bound species shown in Fig. 5 b. At early MISC delays k_entry_ is small. The channel opens, and k_entry_ is large only when sufficient SUB has accumulated in the unit cell (Fig. 2 d). All relevant parameters are listed in Extended Data Table 4.

## Data Availability

Data has been deposited in the Coherent X-ray Imaging Database (CXIDB) with accession code 209. The structure factors and the refined coordinates of XFEL structure of BlaC mixed with sulbactam for 15ms, 30 ms, and 240ms, and the cryo-soaked structure have been deposited in the pDB as entries 8EBI, 8EBR, 8EC4 and 8ECF, respectively.
